# The effects of lipid-lowering therapy on coronary plaque regression: a systematic review and meta-analysis

**DOI:** 10.1038/s41598-021-87528-w

**Published:** 2021-04-12

**Authors:** Yingrui Li, Songbai Deng, Bin Liu, Yulin Yan, Jianlin Du, Yu Li, Xiaodong Jing, Yajie Liu, Jing Wang, Jun Du, Qiang She

**Affiliations:** Department of Cardiology, The Second Affiliated Hospital of Chongqing Medical University, Chongqing, 400016 China

**Keywords:** Cardiology, Cardiovascular diseases

## Abstract

To assess the influence of lipid-lowering therapy on coronary plaque volume, and to identify the LDL and HDL targets for plaque regression to provide a comprehensive overview. The databases searched (from inception to 15 July 2020) to identify prospective studies investigating the impact of lipid-lowering therapy on coronary plaque volume and including quantitative measurement of plaque volume by intravascular ultrasound after treatment. Thirty-one studies that included 4997 patients were selected in the final analysis. Patients had significantly lower TAV (SMD: 0.123 mm^3^; 95% CI 0.059, 0.187; *P* = 0.000) and PAV (SMD: 0.123%; 95% CI 0.035, 0.212; *P* = 0.006) at follow-up. According to the subgroup analyses, TAV was significantly reduced in the LDL < 80 mg/dL and HDL > 45 mg/dL group (SMD: 0.163 mm^3^; 95% CI 0.092, 0.234; *P* = 0.000), and PAV was significantly reduced in the LDL < 90 mg/dL and HDL > 45 mg/dL group (SMD: 0.186%; 95% CI 0.081, 0.291; *P* = 0.001).Thirty-one studies that included 4997 patients were selected in the final analysis. Patients had significantly lower TAV (SMD: 0.123 mm^3^; 95% CI 0.059, 0.187; *P* = 0.000) and PAV (SMD: 0.123%; 95% CI 0.035, 0.212; *P* = 0.006) at follow-up. According to the subgroup analyses, TAV was significantly reduced in the LDL < 80 mg/dL and HDL > 45 mg/dL group (SMD: 0.163 mm^3^; 95% CI 0.092, 0.234; *P* = 0.000), and PAV was significantly reduced in the LDL < 90 mg/dL and HDL > 45 mg/dL group (SMD: 0.186%; 95% CI 0.081, 0.291; *P* = 0.001). Our meta-analysis suggests that not only should LDL be reduced to a target level of < 80 mg/dL, but HDL should be increased to a target level of > 45 mg/dL to regress coronary plaques.

*Trial Registration* PROSPERO identifier: CRD42019146170.

## Introduction

A previous study suggested that the prevalence of coronary heart disease (CHD) resulting in significant myocardial infarction (MI) morbidity and CHD mortality in American adults who are 20 years of age or older was 6.7%^1^. The severity of coronary atherosclerosis in patients with CHD is closely related to adverse cardiovascular events. Therefore, stabilization and regression of coronary atherosclerotic plaques by lipid-lowering therapy plays an important role in the treatment of CHD^2^.

Plaque regression, which includes the removal of lipids and the necrotic core, was shown to restore endothelial function, although the cessation of intravascular smooth muscle cell proliferation is a complex process^3^. Coronary atherosclerotic plaque regression can be detected using various imaging techniques that can measure changes in plaque volume, and intravascular ultrasound (IVUS) is currently one of the most common of such methods^4^. Total atheroma volume (TAV) and percent atheroma volume (PAV) are the indices usually used to evaluate coronary plaque volume. TAV is more sensitive and PAV is more accurate^5^. A plaque has regressed when a reduced plaque volume is detected after treatment. Recent studies have indicated that lipid-lowering therapy can lead to the regression of a coronary atherosclerotic plaque and reduce the incidence of adverse cardiovascular events^6^. A recent meta-regression analysis by Bhindi et al.^7^ showed that a 1% reduction in mean PAV was induced by dyslipidemia therapies and was associated with a 20% reduction in the odds of major adverse cardiac events (MACE).

Statins are the cornerstone of lipid-lowering therapy, but other lipid-lowering drugs include bile acid sequestrants, ezetimibe, and PCSK9 inhibitors^8^. These drugs can reduce blood lipid levels through different mechanisms, including lowering total cholesterol (TC), triglycerides (TG), and low-density lipoproteins (LDL) and increasing high-density lipoproteins (HDL) to some extent. Although there have been a number of meta-analyses concerning lipid-lowering therapy and coronary plaque volume in recent years, most studies have been conservative in the drug interventions selected for inclusion in their studies. For example, they only analyzed TAV but not PAV or they only considered the effect of LDL on plaque regression but not HDL.

Therefore, we performed a meta-analysis to assess the influence of lipid-lowering therapy on coronary plaque volume (TAV and PAV) in this study, and to identify the LDL and HDL targets for plaque regression to provide a comprehensive overview.

## Results

### Selection of sources of evidence

Our search strategy yielded a total of 10,985 studies. There were 4989 studies after the repeated studies were excluded and 885 studies did not meet the inclusion criteria. We then excluded 57 studies because of insufficient data, 32 studies because they were animal studies, and 6 studies because they were duplicate reports of the same study population. Therefore, 31 studies (with 4997 patients in the lipid-lowering therapy group and 769 patients in the control group) that measured TAV or PAV at baseline and follow-up were included in our final analysis^9–39^ (Fig. S1).

### Characteristics of sources of evidence

The main features of the studies are shown in Table 1. The number of patients in each study ranged from 14 to 520. TAV was measured by IVUS in 29 studies^9–19,21–36,38,39^, which had 4761 patients in 52 groups, and PAV was measured by IVUS in 19 studies^10,14,18,20–25,29–34,36–39^, which had 4226 patients in 38 groups.

**Table 1:** Characteristics of included studies.

Study and country	Patient characteristics							Plaque characteristics		Score
	Design	Administration	Participants (n)	Age (years)	Male (%)	LDL-C (mg/dL) BL vs FU	HDL-C (mg/dL) BL vs FU	TAV (mm^3^) BL vs. FU	PAV (%) BL vs. FU	
Okazaki et al.^9^ Japan	Prospective, open-label, randomized, single center study	oral administration	Ato 20 mg/d 24	61.3 ± 10.1	85.7	124.6 ± 34.5 vs 70.0 ± 25.0	45.5 ± 9.9 vs 46.6 ± 10.5	69.6 ± 49.0 vs 61.4 ± 44.9	NS	2
			Control 24	62.5 ± 11.2	85.7	123.9 ± 35.3 vs 119.4 ± 24.6	44.3 ± 11.2 vs 47.4 ± 11.2	59.5 ± 38.6 vs 63.7 ± 40.1		
Nissen et al.^10^ USA	Double-blind, randomized active control multicenter trial	oral administration	Pra 40 mg/d 249	56.6 ± 9.2	73.0	150.2 ± 25.9 vs 110.4 ± 25.8	42.9 ± 11.4 vs 44.6 ± 11.3	194.5 ± 114.8 vs 199.6 ± 112.3	39.5 ± 10.77 vs 41.4 ± 10.0	4
			Ato 80 mg/d 253	55.8 ± 9.8	71.0	150.2 ± 27.9 vs 78.9 ± 30.2	42.3 ± 9.9 vs 43.1 ± 11.3	184.4 ± 115.7 vs 183.9 ± 108.8	38.4 ± 11.27 vs 39.0 ± 10.8	
Tani et al.^11^ Japan	Prospective, single-center, randomized, open trial	oral administration	Pra 5/10/20 mg/d 52	63.0 ± 10.0	75.0	130.0 ± 38.0 vs 104.0 ± 20.0	48.0 ± 11.0 vs 53.0 ± 13.0	47.0 ± 31.0 vs 40.0 ± 25.0	NS	3
			Control 23	62.0 ± 13.0	78.0	123.0 ± 28.0 vs 120.0 ± 30.0	49.0 ± 12.0 vs 47.0 ± 14.0	44.0 ± 18.0 vs 44.0 ± 19.0		
Yokoyama et al.^12^ Japan	Prospective, randomized study	oral administration	Ato 10 mg/d 29	62.1 ± 10.2	90.0	133.0 ± 13.0 vs 87.0 ± 29.0	44.0 ± 11.0 vs 49.0 ± 15.0	69.9 ± 35.0 vs 66.0 ± 32.1	NS	2
			Control 30	64.4 ± 8.7	91.0	NS	NS	55.8 ± 27.5 vs 53.8 ± 25.5		
Kawasaki et al.^13^ Japan	Randomization, open-label, single-center study	oral administration	Ato 20 mg/d 17	66.0 ± 8.7	71.0	155.0 ± 22.0 vs 95.0 ± 15.0	50.0 ± 9.0 vs 56.0 ± 10.0	159.2 ± 31.6 vs 155.4 ± 32.8	NS	2
			Pra 20 mg/d 18	67.0 ± 7.8	72.0	149.0 ± 19.0 vs 102.0 ± 13.0	54.0 ± 12.0 vs 56.0 ± 10.0	166.2 ± 29.5 vs 164.6 ± 34.5		
			Control 17	66.0 ± 6.4	82.0	152.0 ± 20.0 vs 149.0 ± 24.0	50.0 ± 10.0 vs 51.0 ± 11.0	159.0 ± 30.2 vs 159.0 ± 29.5		
Nissen et al.^14^ USA, Canada, Europe, Australia	Prospective, open-label blinded end-points trial	oral administration	Ros 40 mg/d 349	58.5 ± 10.0	70.2	130.4 ± 34.3 vs 60.8 ± 20.0	43.1 ± 11.1 vs 49.0 ± 12.6	212.2 ± 81.3 vs 197.5 ± 79.1	39.6 ± 8.5 vs 38.6 ± 8.5	6
Hong et al.^15^ Korea	Prospective, randomized and comparative study	oral administration	Ros 20 mg/d 16	60.0 ± 8.0	75.0	121.0 ± 45.0 vs 65.0 ± 25.0	52.0 ± 7.0 vs 56.0 ± 13.0	252.0 ± 80.0 vs 246.0 ± 79.0	NS	2
			Ato 40 mg/d 14	62.0 ± 9.0	43.0	127.0 ± 37.0 vs 72.0 ± 26.0	46.0 ± 12.0 vs 49.0 ± 12.0	288.0 ± 98.0 vs 283.0 ± 98.0		
Takayama et al.^16^ Japan	Open-label, multicenter study	oral administration	Ros 20 mg/d 126	62.6 ± 7.7	76.2	140.2 ± 31.5 vs 82.9 ± 18.7	47.1 ± 10.8 vs 55.2 ± 11.7	72.1 ± 38.1 vs 66.8 ± 34.0	NS	6
Nasu et al.^17^ Japan	Prospective, multicenter study	oral administration	Flu 60 mg/d 40	63.0 ± 10.0	80.0	144.9 ± 31.5 vs 98.1 ± 12.7	52.7 ± 12.4 vs 53.9 ± 12.3	440.2 ± 220.3 vs 403.8 ± 209.4	NS	7
			Control 39	62.0 ± 12.0	78.0	122.3 ± 18.9 vs 121.0 ± 21.2	54.3 ± 17.8 vs 54.0 ± 13.9	432.9 ± 247.5 vs 443.7 ± 258.5		
Hiro et al.^18^ Japan	Prospective, randomized, open-label, parallel group study	oral administration	Pit 4 mg/d 125	62.5 ± 11.5	82.4	130.9 ± 33.3 vs 81.1 ± 23.4	45.0 ± 10.1 vs 48.8 ± 12.7	49.8 ± 28.8 vs 41.6 ± 25.0	49.4 ± 10.8 vs 43.7 ± 11.0	3
			Ato 20 mg/d 127	62.4 ± 10.6	81.1	133.8 ± 31.4 vs 84.1 ± 27.4	43.9 ± 9.4 vs 47.1 ± 11.7	63.9 ± 33.9 vs 53.3 ± 31.7	50.5 ± 9.7 vs 44.3 ± 10.7	
Hong et al.^19^ Korea	Prospective, randomized study	oral administration	Sim 20 mg/d 50	58.0 ± 10.0	80.0	119.0 ± 30.0 vs 78.0 ± 20.0	43.0 ± 10.0 vs 48.0 ± 12.0	88.3 ± 26.9 vs 86.3 ± 26.8	NS	2
			Ros 10 mg/d 50	59.0 ± 9.0	74.0	116.0 ± 28.0 vs 64.0 ± 21.0	43.0 ± 11.0 vs 52.0 ± 14.0	91.5 ± 27.5 vs 87.8 ± 27.8		
Nicholls et al.^21^ USA	Prospective, randomized, multicenter, double-blind clinical trial	oral administration	Ato 80 mg/d 519	57.9 ± 8.5	74.4	119.9 ± 28.9 vs 70.2 ± 1.0	44.7 ± 10.7 vs 48.6 ± 0.5	144.2 ± 63.8 vs 138.5 ± 63.2	36.0 ± 8.3 vs 34.9 ± 8.1	5
			Ros 40 mg/d 520	57.4 ± 8.6	72.9	120.0 ± 27.3 vs 62.6 ± 1.0	45.3 ± 11.8 vs 50.4 ± 0.5	144.1 ± 60.8 vs 135.7 ± 57.7	36.7 ± 8.2 vs 35.4 ± 8.2	
Hong et al.^20^ Korea	Prospective, randomized, and comparative study	oral administration	Ros 20 mg/d 65	59.0 ± 10.0	75.0	122.0 ± 37.0 vs 62.0 ± 20.0	47.0 ± 10.0 vs 47.0 ± 12.0	NS	48.0 ± 6.1 vs 47.3 ± 6.5	2
			Ato 40 mg/d 63	58.0 ± 10.0	73.0	117.0 ± 38.0 vs 70.0 ± 24.0	48.0 ± 15.0 vs 47.0 ± 12.0		49.9 ± 6.1 vs 49.7 ± 6.5	
Nozue et al.^23^ Japan	Prospective, open-labeled, randomized, multicenter trial	oral administration	Pit 4 mg/d 58	66.0 ± 9.0	90.0	126.0 ± 28.0 vs 74.0 ± 22.0	46.0 ± 11.0 vs 51.0 ± 13.0	9.1 ± 2.9 vs 8.9 ± 2.8*	55.2 ± 6.1 vs 55.0 ± 6.0	2
			Pra 20 mg/d 61	67.0 ± 11.0	77.0	137.0 ± 35.0 vs 95.0 ± 23.0	47.0 ± 11.0 vs 50.0 ± 12.0	8.8 ± 3.7 vs 8.7 ± 3.6*	53.9 ± 7.8 vs 54.1 ± 7.8	
Kovarnik et al.^22^ Czech Republic	Single, blinded randomized trial	oral administration	Ato + Eze 80 + 10 mg/d 42	63.5 ± 9.3	78.6	3.1 ± 1.3 vs 2.0 ± 0.8#	1.2 ± 0.5 vs 1.2 ± 0.3#	413.9 ± 239.6 vs 401.9 ± 223.1	46.7 ± 6.2 vs 46.3 ± 6.3	4
			Ato 10 mg/d 47	65.1 ± 10.6	66.0	2.7 ± 0.8 vs 2.6 ± 0.8#	1.2 ± 0.3 vs 1.1 ± 0.3#	420.5 ± 189.5 vs 423.3 ± 194.1	46.4 ± 7.0 vs 47.8 ± 8.1	
Guo et al.^26^ China	Prospective, randomized study	oral administration	Ato 10 mg/d 47	62.6 ± 12.0	85.1	3.0 ± 0.7 vs 2.4 ± 0.5#	0.9 ± 0.2 vs 0.9 ± 0.2#	38.1 ± 13.9 vs 38.1 ± 13.6	NS	2
			Ato 20 mg/d 45	59.1 ± 8.5	80.0	2.9 ± 0.6 vs 2.0 ± 0.2#	0.9 ± 0.2 vs 1.0 ± 0.1#	33.8 ± 10.6 vs 36.1 ± 12.0		
			Ato 40 mg/d 43	59.0 ± 12.9	95.3	2.9 ± 0.3 vs 1.9 ± 0.2#	1.0 ± 0.2 vs 1.0 ± 0.2#	37.1 ± 12.0 vs 30.7 ± 8.1		
			Ato 80 mg/d 39	59.0 ± 9.7	87.2	2.8 ± 0.7 vs 1.8 ± 0.3#	0.9 ± 0.1 vs 1.0 ± 0.2#	36.5 ± 14.7 vs 25.0 ± 1.0		
			Control 54	62.1 ± 8.5	88.9	2.9 ± 0.7 vs 3.0 ± 0.6#	1.0 ± 0.2 vs 0.9 ± 0.2#	34.8 ± 13.8 vs 37.5 ± 15.8		
Lee et al.^24^ China	Prospective, randomized, double-blinded study	oral administration	Ato 10 mg/d 19	65.1 ± 1.0	74.0	122.4 ± 39.4 vs 68.5 ± 26.8	41.5 ± 9.5 vs 41.9 ± 10.4	98.5 ± 70.8 vs 94.6 ± 70.6	49.9 ± 7.5 vs 50.2 ± 8.7	5
			Ato 40 mg/d 20	63.7 ± 9.8	90.0	112.4 ± 27.1 vs 52.1 ± 12.6	42.8 ± 17.5 vs 41.5 ± 14.1	144.2 ± 154.5 vs 137.9 ± 144.9	51.6 ± 8.2 vs 50.1 ± 8.3	
Lee et al.^25^ Korea	Prospective, single-center, open-label, randomized comparison trial	oral administration	Ato 20 mg/d 143	57.6 ± 7.6	81.8	110.0 ± 31.0 vs 56.0 ± 18.0	40.0 ± 13.0 vs 47.0 ± 12.0	215.0 ± 89.0 vs 205.0 ± 85.0	42.3 ± 8.6 vs 43.0 ± 8.7	3
			Ros 10 mg/d 128	55.3 ± 9.4	82.8	109.0 ± 31.0 vs 53.0 ± 18.0	40.0 ± 9.0 vs 47.0 ± 11.0	229.0 ± 94.0 vs 210.0 ± 86.0	43.3 ± 9.6 vs 42.3 ± 9.7	
Zhang et al.^27^ China	Open-label, prospective, and randomized clinical trial	oral administration	Ato 80 mg/d 50	64.5 ± 13.8	62.0	105.4 ± 22.7 vs 62.4 ± 16.0	51.5 ± 9.7 vs 58.5 ± 8.9	43.2 ± 6.3 vs 41.7 ± 4.6	NS	2
			Ato 20 mg/d 50	65.5 ± 6.2	58.0	106.1 ± 20.5 vs 80.0 ± 17.8	51.1 ± 9.5 vs 56.6 ± 9.4	42.3 ± 9.3 vs 50.7 ± 9.8		
Hwang et al.^28^ Korea	Prospective, single-center study	oral administration	Ato/Sim/Ros 54	59.0 ± 10.0	70.0	119.7 ± 31.4 vs 67.3 ± 20.4	38.9 ± 8.5 vs 40.1 ± 10.1	76.1 ± 32.1 vs 73.2 ± 31.7	NS	6
Räber et al.^29^ Switzerland	Prospective cohort study	oral administration	Ros 40 mg/d 82	58.5 ± 9.9	92.7	3.3 vs 1.9#	1.1 vs 1.2#	258.3 ± 163.4 vs 245.1 ± 153.0	44.0 ± 10.0 vs 43.0 ± 9.8	8
			Control 21	57.1 ± 12.9	81.0	NS	NS	NS	NS	
Masuda et al.^30^ Japan	Prospective, open-label, randomized, single-center study	oral administration	Ros + Eze 5 + 10 mg/d 21	64.0 ± 7.9	90.5	131.8 ± 25.6 vs 57.3 ± 20.2	53.1 ± 11.8 vs 57.5 ± 15.2	55.3 ± 28.4 vs 47.1 ± 24.6	52.5 ± 12.1 vs 46.9 ± 12.6	3
			Ros 5 mg/d 19	70.2 ± 7.6	84.2	123.0 ± 27.0 vs 75.1 ± 21.4	47.1 ± 12.5 vs 49.1 ± 16.1	43.5 ± 28.5 vs 40.9 ± 24.7	46.4 ± 12.1 vs 45.7 ± 12.6	
Tsujita et al.^31^ Japan	Prospective, randomized, controlled, assessor-blind, multicenter study	oral administration	Ato + Eze 100	66.0 ± 10.0	78.0	109.8 ± 25.4 vs 63.2 ± 16.3	41.1 ± 9.5 vs 45.6 ± 11.9	72.6(37.6,117.4) vs 69.6(35.0,107.2)	51.3 ± 10.8 vs 49.3 ± 10.3	3
			Ato 102	67.0 ± 10.0	78.0	108.3 ± 26.3 vs 73.3 ± 20.3	40.0 ± 10.3 vs 43.3 ± 11.5	76.3(45.5,128.4) vs 77.3(45.4,126.2)	50.9 ± 11.4 vs 50.4 ± 11.6	
Matsushita et al.^32^ Japan	Prospective, randomized, and comparative study	oral administration	Ato 20 mg/d 26	62.4 ± 8.7	92.0	135.0 ± 27.0 vs 72.0 ± 22.0	43.0 ± 10.0 vs 48.0 ± 15.0	70.3 ± 5.8 vs 63.0 ± 22.7	50.2 ± 2.3 vs 46.6 ± 12.6	2
			Pit 4 mg/d 26	62.8 ± 11.4	85.0	140.0 ± 20.0 vs 78.0 ± 13.0	50.0 ± 13.0 vs 50.0 ± 13.0	62.5 ± 5.8 vs 57.4 ± 36.4	44.1 ± 2.3 vs 41.2 ± 14.5	
			Pra 10 mg/d 25	63.6 ± 8.6	72.0	152.0 ± 30.0 vs 107.0 ± 23.0	51.0 ± 12.0 vs 54.0 ± 12.0	74.5 ± 5.9 vs 75.7 ± 33.1	46.0 ± 2.3 vs 47.5 ± 13.8	
			Flu 30 mg/d 25	62.4 ± 12.2	72.0	139.0 ± 29.0 vs 103.0 ± 29.0	48.0 ± 16.0 vs 50.0 ± 15.0	56.2 ± 5.9 vs 55.0 ± 25.5	44.7 ± 2.3 vs 45.1 ± 10.8	
Takayama et al.^35^ Japan	Prospective, open-label, randomized, investigator-blinded, parallel-comparison study	oral administration	Ros 20 mg/d 18	65.1 ± 10.1	72.0	130.3 ± 25.5 vs 61.7 ± 16.5	45.3 ± 9.7 vs 47.7 ± 9.3	56.5 ± 34.2 vs 53.4 ± 32.3	NS	4
			Ros 2.5 mg/d 19	63.8 ± 8.5	83.0	130.9 ± 28.5 vs 89.7 ± 29.0	44.6 ± 13.0 vs 47.7 ± 14.4	58.1 ± 33.5 vs 59.3 ± 31.7		
Oemrawsingh et al.^33^ The Netherlands	Prospective, investigator-initiated, single-centre study	oral administration	Ros 40 mg/d 164	60.4 (55.3, 65.9)	84.1	2.49 ± 0.85 vs 1.73 ± 0.71#	1.11 ± 0.31 vs 1.23 ± 0.37#	243.9 ± 151.3 vs 247.8 ± 148.6	40.7 ± 10.2 vs 41.6 ± 9.7	8
			Control 77	57.5 (51.6, 66.0)	79.2	NS	NS	NS	NS	
Nicholls et al.^34^ Australia	Multicenter, double-blind, placebo-controlled, randomized clinical trial	subcutaneous injection	Evo 420 mg/d 484	59.8 ± 9.6	72.1	92.6(90.1,95.0) vs 36.6(34.5,38.8)	46.7(45.5,47.8) vs 51.0(49.8,52.1)	187.0(199.1,194.8) vs 181.5(174.1,188.9)	36.4(35.6,37.2) vs 35.6(34.8,36.4)	5
			Control 484	59.8 ± 8.8	72.3	92.4(90.0,94.8) vs 93.0(90.5,95.4)	45.4(44.2,46.5) vs 47.1(46.0,48.2)	191.4(183.2,199.6) vs 190.6(182.5,198.7)	37.2(36.4,38.0) vs 37.3(36.5,38.1)	
Ueda et al.^37^ Japan	Multicenter, prospective, randomized,open-label, blinded-endpoint trial	oral administration	Ato (10–20)mg/d 54	68.0 ± 11.0	81.0	100.0 ± 27.0 vs 75.0 ± 16.0	45.0 ± 9.0 vs 45.0 ± 11.0	NS	48.5 ± 10.2 vs 48.2 ± 10.4	3
			Ato + Eze (10–20) + 10 mg/d 54	71.0 ± 8.0	76.0	101.0 ± 27.0 vs 61.0 ± 17.0	46.0 ± 18.0 vs 44.0 ± 12.0		50.0 ± 9.8 vs 49.3 ± 9.8	
Hougaard et al.^36^ Denmark	Single-center double blinded randomized trial	oral administration	Ato + Eze 80 + 10 mg/d 39	53.3 ± 11.0	90.7	3.7 ± 0.7 vs 1.4 ± 0.8#	1.1 ± 0.3 vs 1.1 ± 0.3	200.0(135.6,311.9) vs 189.3(126.4,269.1)	40.1 ± 8.6 vs 39.2 ± 9.0	5
			Ato 80 mg/d 41	57.2 ± 9.1	81.8	4.1 ± 0.9 vs 2.0 ± 0.5#	1.1 ± 0.3 vs 1.1 ± 0.3	218.4(163.5,307.9) vs 212.2(149.9,394.8)	43.3 ± 9.4 vs 42.2 ± 10.7	
Hibi et al.^38^ Japan	Prospective, randomized open-label parallel group study	oral administration	Pit + Eze 2 + 10 mg/d 50	65.0 ± 10.0	82.0	123.0 ± 32.0 vs 64.0 ± 18.0	45.0 ± 14.0 vs 49.0 ± 12.0	233.0 ± 175.0 vs 222.0 ± 17.5	44.3 ± 9.4 vs 42.9 ± 9.6	3
			Pit 2 mg/d 53	63.0 ± 12.0	77.0	126.0 ± 33.0 vs 87.0 ± 21.0	46.0 ± 11.0 vs 49.0 ± 15.0	251.0 ± 155.0 vs 240.0 ± 153.0	43.9 ± 10.6 vs 42.0 ± 10.0	
Thondapa et al.^39^ USA	Prospective single-center randomized clinical trial	oral administration	Ros 10 mg/d 24	57.5	58.0	100.0 ± 21.0 vs 76.0 ± 34.0	51.0 ± 15.0 vs 52.0 ± 13.0	109.2 ± 62.1 vs 102.5 ± 62.2	52.5 ± 9.2 vs 51.3 ± 8.1	2
			Ato 20 mg/d 19	54.2	68.0	115.0 ± 28.0 vs 80.0 ± 32.0	50.0 ± 12.0 vs 50.0 ± 18.0	83.3 ± 48.5 vs 77.9 ± 48.6	54.5 ± 9.5 vs 54.4 ± 9.5	

*LDL-C* low-density lipoprotein cholesterol, *HDL-C* high–density lipoprotein cholesterol, *TAV* total atheroma volume, *PAV* percentage atheroma volume, *BL* baseline, *FU* follow-up, *Ato* atorvastatin, *Ros* rosuvastatin, *Pra* pravastatin, *Pit* pitavastatin, *Sim* simvastatin, *Flu* fluvaststin, *Eze* ezetimibe, *Evo* evolocumab, ^#^the value was provided as mmol/l; *the value was provided as volume index defined as the volume divided by the segment length (mm^3^/mm).

### Critical appraisal within sources of evidence

The quality of randomized controlled trials was assessed by the Jadad quality scale, and the quality of non-randomized controlled trials was evaluated by the Newcastle Ottawa scale (NOS). The details were shown in Table S1 and S2.

Publication bias can influence the results of a meta-analysis. Therefore, a funnel plot and Egger’s and Begg’s tests were used to evaluate the potential publication bias in the included studies. The assessment of the symmetry of the funnel plots for the TAV or PAV showed little publication bias in our results (Fig. S2). Egger’s test (TAV: *P* = 0.315; PAV: *P* = 0.272) and Begg’s test (TAV: *P* = 0.398; PAV: *P* = 0.209) both confirmed this finding.

A sensitivity analysis was conducted by performing additional meta-analyses after deleting individual studies one by one. The results of the sensitivity analysis showed that none of the studies influenced the pooled SMD, which indicated that our meta-analysis was statistically stable (Fig. S3).

### Results of individual sources of evidence

The relevant data of each included study were presented in Table 1.

### Synthesis of results

A total of 29 studies reported that TAV was significantly reduced in patients at follow-up (SMD: 0.123 mm^3^; 95% CI 0.059, 0.187; *P* < 0.001). There was heterogeneity among the studies (I^2^ = 47.0%, *P* < 0.001). A total of 18 studies reported a significant reduction in PAV of patients at follow-up (SMD: 0.123%; 95% CI 0.035, 0.212; *P* = 0.006). There was heterogeneity among the studies (I^2^ = 69.3%, *P* < 0.001).

**Figure 1:**
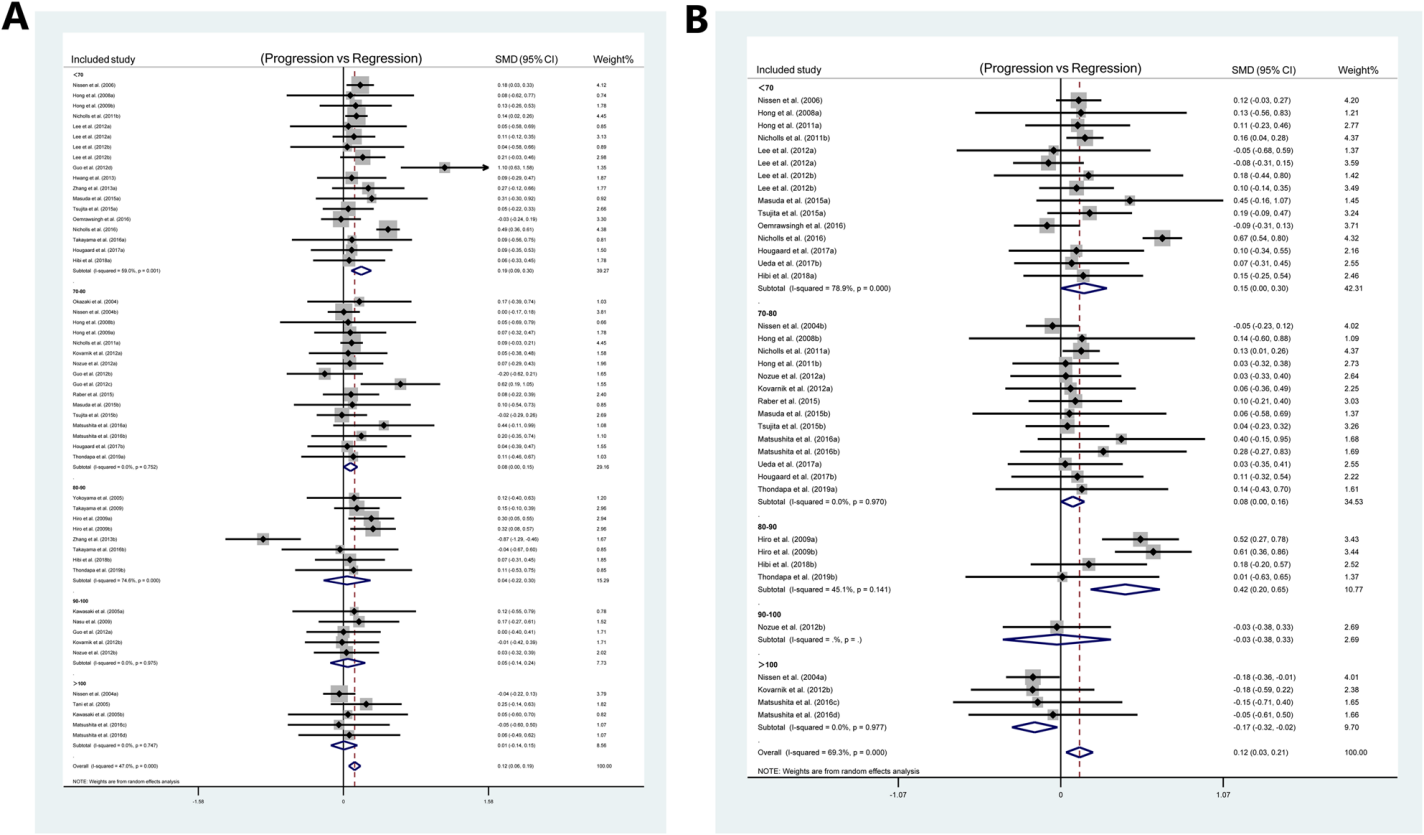
Subgroup analysis for SMD in plaque volume between patients at baseline and follow-up: (**A**) subgroup analysis of TAV according to the different levels of LDL; (**B**) subgroup analyses of PAV according to the different levels of LDL.

To explore the target level of LDL for plaque regression, the included studies were divided into five groups according to the levels of LDL at follow-up: < 70, 70–80, 80–90, 90–100, > 100 mg/dL. The subgroup analysis of TAV data showed significant plaque regression in the LDL < 70 mg/dL group (SMD: 0.195 mm^3^; 95% CI 0.086, 0.304; *P* < 0.001) (I^2^ = 59.0%, *P* = 0.001, Fig. 1A) and the 70–80 mg/dL group (SMD: 0.078 mm^3^; 95% CI 0.003, 0.153; *P* = 0.042) (I^2^ = 0.0%, *P* = 0.752, Fig. 1A) at follow-up. The subgroup analysis of PAV data showed significant plaque regression in the LDL < 70 mg/dL group (SMD: 0.152%; 95% CI 0.001, 0.303; *P* = 0.049) (I^2^ = 78.9%, *P* < 0.001, Fig. 1B), 70–80 mg/dL group (SMD: 0.079%; 95% CI 0.003, 0.155; *P* = 0.042) (I^2^ = 0.0%, *P* = 0.97, Fig. 1B) and LDL 80–90 mg/dL group (SMD: 0.423%; 95% CI 0.196, 0.651; *P* < 0.001) (I^2^ = 45.1%, *P* = 0.141, Fig. 1B) at follow-up. The total effect was statistically significant.

**Figure 2:**
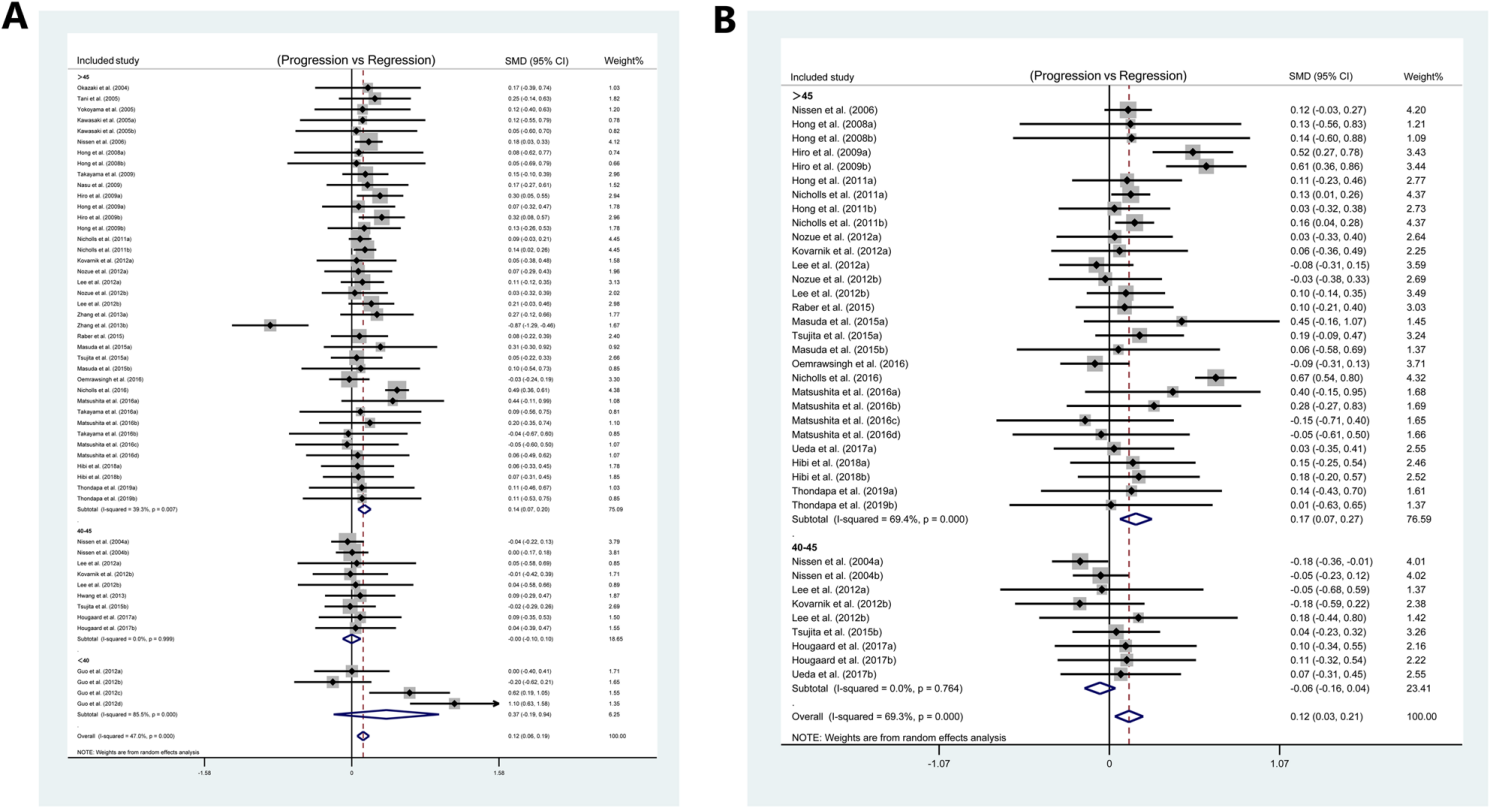
Subgroup analysis for SMD in plaque volume between patients at baseline and follow-up: (**A**) subgroup analysis of TAV according to the different levels of HDL; (**B**) subgroup analyses of PAV according to the different levels of HDL.

In order to identify the target level of HDL for plaque regression, the included studies were divided into three groups according to the levels of HDL at follow-up: > 45, 40–45, < 40 mg/dL. The subgroup analysis of TAV data showed significant plaque regression in the HDL > 45 mg/dL group (SMD: 0.137 mm^3^; 95% CI 0.068, 0.205; *P* < 0.001) (I^2^ = 39.3%, *P* = 0.007, Fig. 2A). Meanwhile, the subgroup analysis of PAV data also showed significant plaque regression in the HDL > 45 mg/dL group (SMD: 0.166%; 95% CI 0.066, 0.266; *P* = 0.001) (I^2^ = 69.4%, *P* < 0.001, Fig. 2B), and the total effect was statistically significant.

**Figure 3:**
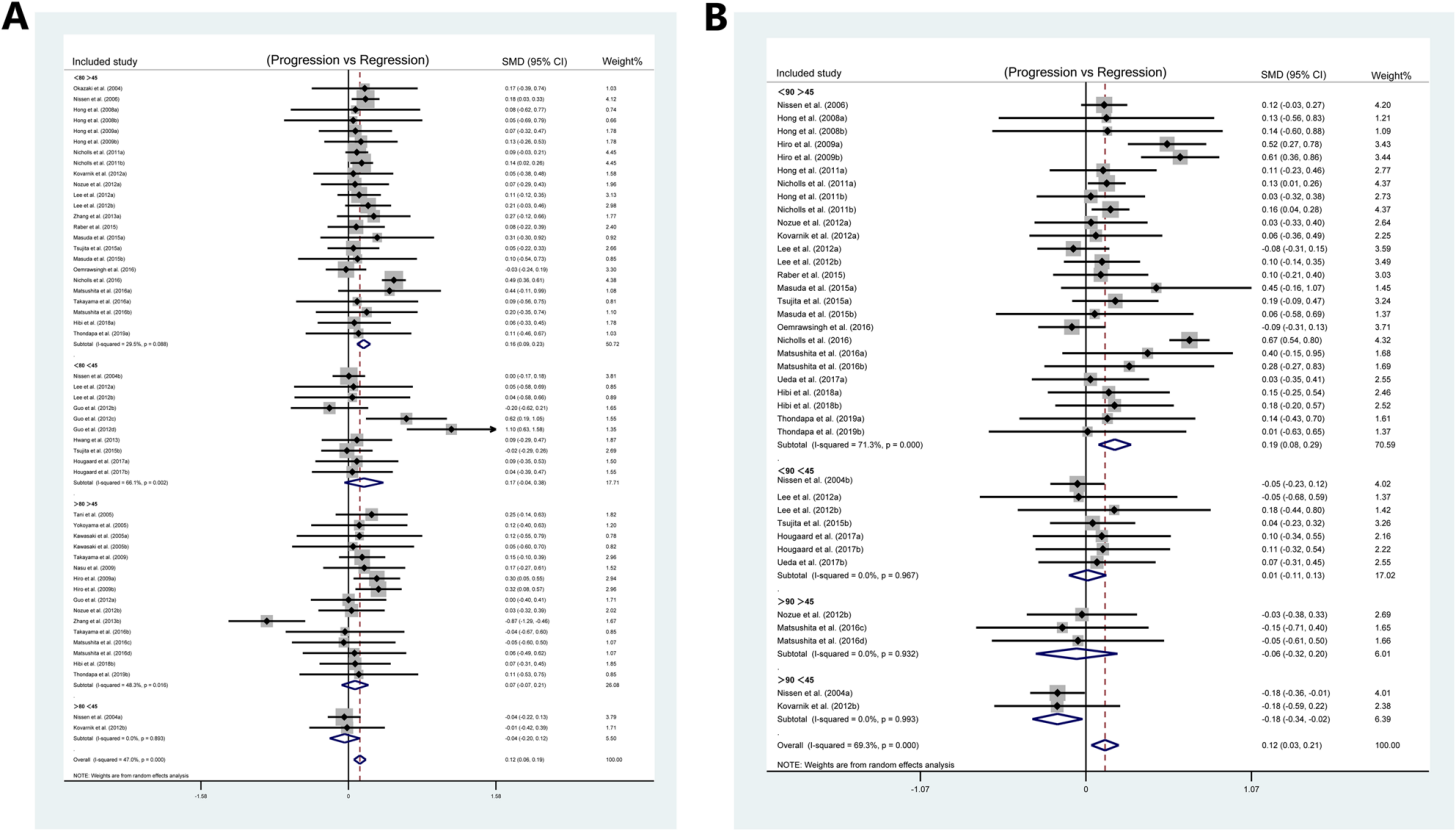
Subgroup analysis for SMD in plaque volume between patients at baseline and follow-up: (**A**) subgroup analysis of TAV according to the different levels of LDL and HDL; (**B**) subgroup analyses of PAV according to the different levels of LDL and HDL.

To explore the combined effects of LDL reduction and HDL incrementation on plaque regression, the studies concerning TAV were divided into four groups according to the above findings: LDL < 80 mg/dL and HDL > 45 mg/dL group, LDL < 80 mg/dL and HDL < 45 mg/dL group, LDL > 80 mg/dL and HDL > 45 mg/dL group, and LDL > 80 mg/dL and HDL < 45 mg/dL group. In the meantime, we also divided the studies concerning PAV into four groups: LDL < 90 mg/dL and HDL > 45 mg/dL group, LDL < 90 mg/dL and HDL < 45 mg/dL group, LDL > 90 mg/dL and HDL > 45 mg/dL group, LDL > 90 mg/dL and HDL < 45 mg/dL group. There was a significant plaque regression in the LDL < 80 mg/dL and HDL > 45 mg/dL group (SMD: 0.163 mm^3^; 95% CI 0.092, 0.234; *P* < 0.001) (I^2^ = 29.5%, *P* = 0.088, Fig. 3A) in the subgroup analysis of TAV, and there was a significant plaque regression in the LDL < 90 mg/dL and HDL > 45 mg/dL group (SMD: 0.186%; 95% CI 0.081, 0.291; *P* = 0.001) (I^2^ = 71.3%, *P* < 0.001, Fig. 3B) in the subgroup analysis of PAV. The total effect was statistically significant.

A subgroup analysis of the administration of different drugs was conducted to eliminate the significant heterogeneity among the studies. The subgroup analysis concerning TAV indicated there was a significant decrease in heterogeneity in the oral administration group (SMD: 0.105 mm^3^; 95% CI 0.051, 0.159; *P* < 0.001) (I^2^ = 23.5%, *P* = 0.071, Fig. S4A) and subcutaneous injection group (SMD: 0.487 mm^3^; 95% CI 0.359, 0.614; *P* < 0.001, Fig. S4A). In addition, the heterogeneity also showed a significant decrease in the subgroup analysis concerning PAV in the oral administration group (SMD: 0.096%; 95% CI 0.033, 0.159; *P* = 0.003) (I^2^ = 33.1%, *P* = 0.028, Fig. S4B) and subcutaneous injection group (SMD: 0.667%; 95% CI 0.537, 0.796; *P* < 0.001, Fig. S4B). The total effect was statistically significant.

**Figure 4:**
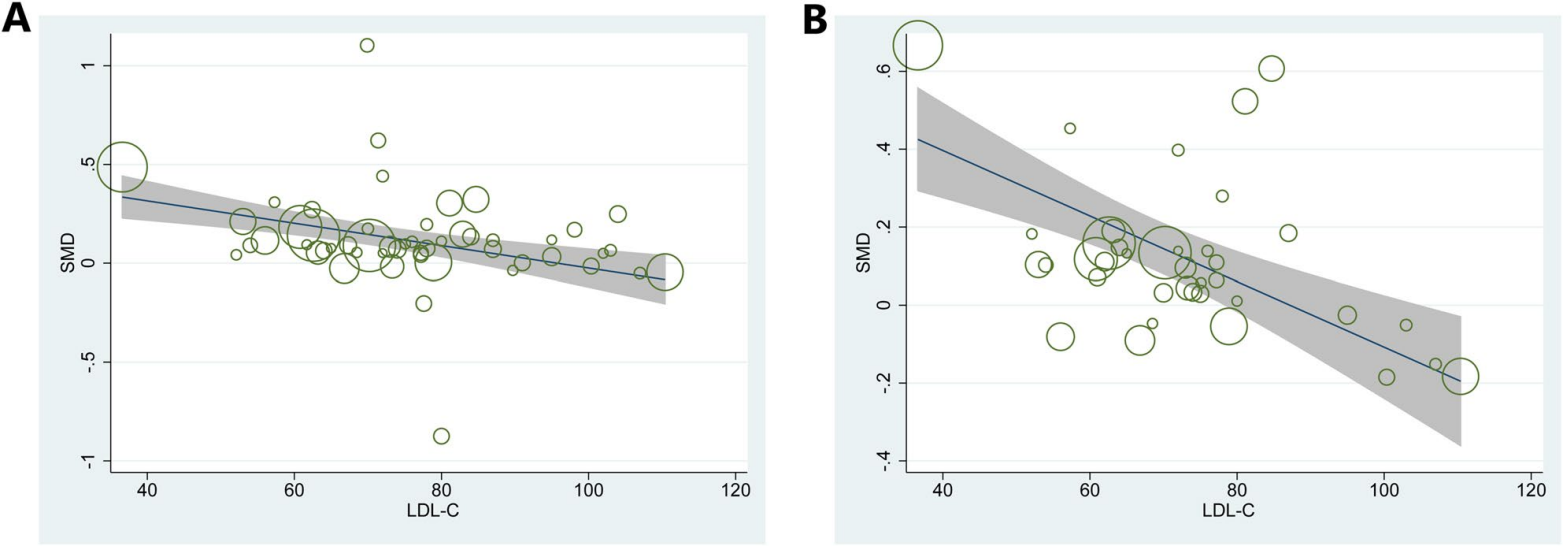
Meta–regression analyses for SMD in plaque volume between patients at baseline and follow-up: (**A**) effect of LDL on TAV; (**B**) effect of LDL on PAV.

A regression analysis was performed to assess other potential factors that may have influenced the outcomes. Our analysis indicated that LDL levels at follow-up significantly influenced TAV and PAV (TAV: *P* = 0.011, tau^2^ = 0.0112, Adj R-squared = 43.98%, I-squared res = 30.49%; PAV: *P* = 0.016, tau^2^ = 0.0244, Adj R-squared = 24.43%, I-squared res = 51.73%, Fig. 4). At the same time, gender significantly affected TAV (*P* = 0.035, tau^2^ = 0.0195, Adj R-squared = 1.43%, I-squared res = 46.56%). The dosage of drugs (*P* = 0.04, tau^2^ = 0.0269, Adj R-squared = 19.52%, I-squared res = 53.84%) and TG levels at baseline (*P* = 0.04, tau^2^ = 0.0263, Adj R-squared = 24.95%, I-squared res = 59.11%) significantly affected PAV. Other factors, including age, region, drugs, smoking, diabetes, hypertension, and blood lipid levels (HDL, TC), did not influence the results. The details of regression analysis outcomes were shown in Table S3 and S4.

## Discussion

A total of 31 studies with 4997 enrolled patients who received lipid-lowering therapy were included in our meta-analysis. The changes in coronary plaque volume were measured by IVUS, and the results showed significant coronary plaque regression in patients after receiving lipid-lowering therapy. The subgroup analysis indicated that TAV was significantly reduced when LDL at follow-up was less than 80 mg/dL and HDL was greater than or equal to 45 mg/dL, and PAV was significantly decreased when LDL at follow-up was less than 90 mg/dL and HDL was greater than or equal to 45 mg/dL. These findings were also confirmed by sensitivity analysis. Regression analysis showed that LDL levels at follow-up significantly influenced our results.

To better understand the link between lipid-lowering therapy and plaque regression, a meta-analysis was conducted to explore the changes in TAV and PAV in patients after receiving treatment, and the results showed a significant reduction in TAV and PAV at follow-up with some heterogeneity. We performed a subgroup analysis of the different types of drug administration in patients to explore the source of heterogeneity. The heterogeneity in the subgroups decreased significantly, suggesting that different drug regimens may be potential sources of heterogeneity.

In recent studies, LDL has been shown to accumulate abnormally in the vascular wall due to the dysfunction of endothelial cells. Moreover, LDL can be converted into ox-LDL, which can damage endothelial cells and smooth muscle cells, thereby causing abnormal activation of the endothelial cells, producing foam cells and eventually promoting plaque progression^40^. According to the latest guideline for the management of blood cholesterol and dyslipidemias, experts recommended that patients with a very high risk of arteriosclerotic cardiovascular disease (ASCVD) reduce LDL levels to below 70 mg/dL, which can delay the progress of risk factors and reduce the incidence of adverse events^8^. Our subgroup analysis showed that TAV was significantly reduced when the LDL levels were less than 80 mg/dL at follow-up, and PAV showed a significant decrease when the LDL levels were less than 90 mg/dL at follow-up.

In previous studies, HDL was shown to play an important role in the regression of coronary plaque by reverse cholesterol transport (RCT)^3^. HDL is mainly synthesized by apoAI and apoAII, which can clear or reuse cholesterol through lipid metabolism pathways, thereby reducing the progressive accumulation of cholesterol in plaque and promoting the regression of plaque^41^. A rise in HDL levels can reduce the incidence of cardiovascular adverse events. In the latest guideline for the management of dyslipidemias, HDL is the class I recommendation for lipid analyses in cardiovascular disease risk estimation^42^. In recent research, a rise in HDL level was shown to promote regression of coronary plaque and reduce the occurrence of MACE when LDL was greater than or equal to 70 mg/dL in patients receiving statin therapy^43^. Our subgroup analysis demonstrated a significant reduction in both TAV and PAV when HDL levels were greater than or equal to 45 mg/dL after lipid-lowering therapy.

Plaque regression is affected by various factors. In a study by Nicholls et al., a rise in HDL and reduction in TG slowed the progression of coronary atherosclerotic plaque^44^. A previous study also suggested that diabetes and hypertension can damage vascular endothelial function and promote the progression of coronary atherosclerotic plaque^45^. Changes in plaque volume at follow-up can also be affected by factors such as drug dose, method of observation, and location of the plaque. Therefore, we conducted a meta-regression analysis to assess other factors that could influence outcomes. The results indicated that LDL at follow-up affected both TAV and PAV, and gender only affected TAV, while dose of drugs and TG levels at baseline only affected PAV. Other risk factors did not influence the results.

Our analysis suggests that patients with CHD require an LDL level below 80 mg/dL and HDL above 45 mg/dL at follow-up for regression of coronary plaques to occur. TAV and PAV exhibited different target levels of LDL for plaque regression in our analysis, which may be due to differences in the number of studies included. The results have considerable significance for current CHD patient management and further research on coronary plaque regression. A recent study suggested that the regression of coronary atherosclerotic plaque in patients with stable CHD is closely related to myocardial infarction and vascular revascularization, but not significantly associated with MACE^46^. However, in the study by Hirohata et al., patients with atheroma progression displayed more adverse events than patients with no progression^47^. Therefore, combined with our research, these results suggest that for patients with CHD controlling LDL at follow-up below 80 mg/dL and HDL above 45 mg/dL can have a positive effect, improving patient prognosis. At the same time, the regression analysis also suggests the important role of LDL in plaque regression, which can provide new ideas for research on plaque regression in the future.

## Limitations

This study also had several potential limitations. Most importantly, some of the studies included in the meta-analysis had a small sample size. Furthermore, some subgroup analysis included limited studies; therefore, more studies are needed to support the results. Finally, it is important to assess heterogeneity among studies, and although it may not be possible to identify all possible sources of heterogeneity, the stability of our outcomes was confirmed after adjusting for potential publication bias.

## Conclusions

In general, recent meta-analyses have only considered the effect of LDL on plaque regression, whereas our meta-analysis indicates not only that LDL should be reduced to a target level of < 80 mg/dL, but also that HDL should be increased to a target level of > 45 mg/dL to regress coronary plaque.

## Methods

This meta-analysis strictly abided by the PRISMA guidelines^48^.

### Protocol and registration

The review protocol was developed according to PRISMA guidelines , and was registered in PROSPERO. The registration number was CRD42019146170.

### Eligibility criteria

Studies were included if they met the following inclusion criteria: (1) the study design was a prospective clinical cohort study; (2) the impact of lipid-lowering therapy on coronary plaque volume was investigated, including quantitative measurement of plaque volume by IVUS; (3) sufficient information on blood lipids and IVUS findings at baseline and at the end of the study were presented; (4) lipid-lowering therapy was administered for at least 6 months; and (5) primary or secondary outcomes included change in total atheroma volume or percent atheroma volume.

Studies were excluded if they were: (1) non-clinical studies, observational studies, or retrospective studies; (2) duplicate reports or secondary or post hoc analyses of the same study population; or they contained (3) insufficient information on plaque volume and blood lipids (mean, SD, and sample sizes).

### Information sources

The review searched studies based on PICOS (populations: CHD patients; interventions: lipid-lowering therapy; comparisons: before lipid-lowering therapy; outcomes: change in plaque volume as the first or secondary outcome; study design: prospective clinical cohort study) strategy in online databases (PubMed, EMBASE, Cochrane Library, and Web of Science) up to 15 July 2020 were systematically searched.

### Search

The following search terms were searched in databases: (intravascular ultrasound OR IVUS) AND (lipid-lowering OR PCSK9 inhibitor OR PCSK9 inhibitors OR evolocumab OR alirocumab OR cholesterol absorption inhibitor OR cholesterol absorption inhibitors OR ezetimibe OR statin OR statins OR rosuvastatin OR pravastatin OR fluvastatin OR simvastatin OR atorvastatin OR pitavastatin OR lovastatin OR cerivastatin OR hydroxymethylglutaryl-CoA reductase inhibitors OR bile acid sequestrants) AND (plaque OR plaque, atherosclerotic). This analysis only included human studies and those published in English.

### Selection of sources of evidence

Two reviewers (Yingrui Li and Songbai Deng) extracted data from included studies independently. When there was a disagreement on studies, the two reviewers reached a consensus through negotiation. The data extracted from each study included the sample size, LDL, HDL, TAV, and PAV at baseline and at the end of the study.

### Data charting process

The Microsoft Excel was applied for a data charting form in this study. One single reviewer tested the form via 10 full-text articles. Then, both reviewers modified the form and confirmed the details of the process and data obtaining. None of reviewers found a need for additional modifications to the form.

### Data items

Data about article (title, authors, year, area and study design), participant characteristics (sample size, age, BMI, gender, PAV, TAV, HDL, LDL, drug administration, smoking, diabetes and hypertension), and information on the assessment used (included population, methods for measuring participation and measurement of exposure factors) were extracted from included studies.

### Critical appraisal of individual sources of evidence

The Jadad quality scale was used to assess the quality of randomized controlled trials, and the NOS was used to assess the quality of non-randomized controlled trials. The results ranged from 0 to 5 and 0 to 9, respectively, with higher scores representing better methodology quality (Table 1).

### Synthesis of results

To calculate the 95% CI of the pooled standard mean difference (SMD) or weighted mean difference (WMD), we used a fixed effects model or a random effects model to perform all statistical analyses using Stata 12.0. *P* values < 0.05 were considered statistically significant and all *P* values were two-sided. The χ^2^ and I^2^ statistics were used to evaluate the heterogeneity between studies. If *P* was < 0.1 and I^2^ was > 30%, a random effects model was used; otherwise, a fixed effects model was used. Considering that one of the purposes of this study was to identify the LDL and HDL targets for plaque regression and determine the potential impact of confounding factors on the results of the study, we performed subgroup analyses of drug administration regimens and LDL and HDL levels at follow-up. Meanwhile, to examine the influence of individual studies on the total merged effects, we used a sensitivity analysis to evaluate the stability of the results. We applied Begg’s and Egger’s tests to assess publication bias in the included studies, and we assessed possible small sample effects by analyzing the symmetry of a funnel plot. *P* values < 0.10 were considered statistically significant^49,50^. Taking into account the differences between the studies, all of our analyses used a random effects model.

To explore the link between the dependent variable and the covariate, meta-regression is often used. We hypothesized that the included studies may have shown differences according to the age, gender, region, drugs and drug dosages, smoking, diabetes, hypertension, and blood lipids (LDL, HDL, TC, TG) of the patients. To evaluate the possible impact of these factors on the results of the meta-analysis, we established a regression model with the TAV or PAV value as the dependent variable (y) and the abovementioned covariate as the independent variable (x).

### Patient and public involvement

Patients were not involved in the design or conduct of the study.

## Supplementary Information

Supplementary Information 1.

Supplementary Figure S1.

Supplementary Figure S2.

Supplementary Figure S3.

Supplementary Figure S4.

## Data Availability

No datasets were generated or analysed during the current study.
