# Supplementary material for: The effects of lipid-lowering therapy on coronary plaque regression: a systematic review and meta-analysis
Source: Sci Rep. 2021 Apr 12;11:7999. doi: 10.1038/s41598-021-87528-w (PMC8042107; doi:10.1038/s41598-021-87528-w)
Supplement: Supplementary file 1 — Supplementary Information 1. [file 41598_2021_87528_MOESM1_ESM.docx]

**Supplement** **Material**

**The effects of lipid-lowering therapy on coronary plaque regression: a systematic review and meta-analysis**

**Authors:** Yingrui Li^#1^, MD; Songbai Deng^#1^, PhD; Bin Liu^1^, PhD; Yulin Yan^1^, PhD; Jianlin Du^1^, Prof ; Yu Li^1^, PhD; Xiaodong Jing^1^, PhD; Yajie Liu^1^, PhD; Jing Wang^1^, MD; Jun Du^1^, MD; Qiang She^1*^, Prof

**Affiliation:** 1 Department of Cardiology, The Second Affiliated Hospital of Chongqing Medical University, Chongqing, 400016, China.

^#^ Yingrui Li and Songbai Deng contributed equally to this work

* Corresponding author. Department of Cardiology, The Second Affiliated Hospital of Chongqing Medical University, Chongqing, 400016, China.

Tel: 86 23 63693753 (Q.she)

E-mail address: 1065269334@qq.com(Yingrui Li); [673011271@qq.com(Songbai](mailto:673011271@qq.com(Songbai) Deng); [475264798@qq.com(Bin](mailto:475264798@qq.com(Bin) Liu)；[691011163@qq.com(Yulin](mailto:691011163@qq.com(Yulin) Yan); [397030100@qq.com(Jianlin](mailto:397030100@qq.com(Jianlin) Du); [yanyan0985@126.com](mailto:yanyan0985@126.com)(Yu Li)；[1198227565@qq.com](mailto:1198227565@qq.com)(Xiaodong Jing)；[516374872@qq.com(Yajie](mailto:516374872@qq.com(Yajie) Liu); [729932339@qq.com(Jing](mailto:729932339@qq.com(Jing) Wang); [1198822749@qq.com(Jun](mailto:1198822749@qq.com(Jun) Du); qshe98@cqmu.edu.cn (Q. She).

**Table S1. Jadad Quality Scale Evaluation of Study Quality**

| **STUDY** | **RANDOMISATION** | **BLINDING** | **WITHDRAWALS AND DROPOUTS** |
| --- | --- | --- | --- |
| **Okazaki et al.**  **(2004)** | ***** | **NA** | ***** |
| **Nissen et al.**  **(2004)** | ****** | ***** | ***** |
| **Tani et al.**  **(2005)** | ***** | ***** | ***** |
| **Yokoyama et al.**  **(2005)** | ***** | **NA** | ***** |
| **Kawasaki et al.**  **(2005)** | ***** | **NA** | ***** |
| **Hong et al.**  **(2008)** | ***** | **NA** | ***** |
| **Hiro et al.**  **(2009)** | ***** | ***** | ***** |
| **Hong et al.**  **(2009)** | ***** | **NA** | ***** |
| **Nicholla et al.**  **(2011)** | ****** | ****** | ***** |
| **Hong et al.**  **(2011)** | ***** | **NA** | ***** |
| **Nozue et al.**  **(2012)** | ***** | **NA** | ***** |
| **Kovarnik et al.**  **(2012)** | ****** | ***** | ***** |
| **Guo et al.**  **(2012)** | ***** | **NA** | ***** |
| **Lee et al.**  **(2012) (China)** | ****** | ****** | ***** |
| **Lee et al.**  **(2012)** **(Korea)** | ****** | **NA** | ***** |
| **Zhang et al.**  **(2013)** | ***** | **NA** | ***** |
| **Masuda et al.**  **(2015)** | ****** | **NA** | ***** |
| **Tsujita et al.**  **(2015)** | ***** | ***** | ***** |
| **Matsushita et al.**  **(2016)** | ***** | **NA** | ***** |
| **Takayama et al.**  **(2016)** | ***** | ****** | ***** |
| **Nicholls et al.**  **(2016)** | ****** | ****** | ***** |
| **Ueda et al.**  **(2017)** | ***** | ***** | ***** |
| **Hougaard et al.**  **(2017)** | ****** | ****** | ***** |
| **Hibi et al.**  **(2018)** | ***** | ***** | ***** |
| **Thondapa et al.**  **(2019)** | ***** | **NA** | ***** |

**The Jadad quality scale evaluates the included studies based on randomisation, blinding and withdrawals and dropouts. The maximum score for each criteria is 2, 2 and 1, respectively, with the maximum total score equaling 5.** **NA: not applicable.**

**Table S2. Newcastle-Ottawa Scale (NOS) Evaluation of Study Quality**

| **STUDY** | **SELECTION** | **COMPARABILITY** | **EXPOSURE FACTORS** |
| --- | --- | --- | --- |
| **Nissen et al.**  **(2006)** | ******* | ***** | ****** |
| **Takayama et al.**  **(2009)** | ******* | ****** | ***** |
| **Nasu et al.**  **(2009)** | ******** | ****** | ***** |
| **Hwang et al.**  **(2013)** | ******* | ***** | ****** |
| **Raber et al.**  **(2015)** | ******** | ****** | ****** |
| **Oemrawsingh et al.**  **(2016)** | ******** | ****** | ****** |

**The Newcastle-Ottawa Scale (NOS) evaluates the included studies based on selection, comparability and outcome. The maximum score for each criteria is 4, 2 and 3, respectively, with the maximum total score equaling 9.**

**Table S3. Regression Analysis for PAV**

| **Variable** | **Coefficient** | **95% CI** | **tau^2^** | **Adj R-squared (%)** | **I-squared res (%)** | **P** |
| --- | --- | --- | --- | --- | --- | --- |
| **Age** | **0.0033** | **-0.0166, 0.0233** | **0.033** | **-2.30** | **69.50** | **0.74** |
| **Gender** | **0.0038** | **-0.0070, 0.0147** | **0.033** | **-2.75** | **70.09** | **0.48** |
| **Region** | **0.0194** | **-0.0728, 0.1116** | **0.035** | **-2.43** | **68.12** | **0.67** |
| **LDL at baseline** | **-0.0021** | **-0.0071, 0.0028** | **0.031** | **62.01** | **3.33** | **0.39** |
| **LDL at follow-up** | **-0.0059** | **-0.0106, -0.0011** | **0.024** | **24.43** | **51.73** | **0.02** |
| **HDL at baseline** | **0.0135** | **-0.0147, 0.0416** | **0.031** | **4.33** | **64.68** | **0.34** |
| **HDL at follow-up** | **0.0241** | **-0.0008, 0.0491** | **0.027** | **17.86** | **59.46** | **0.06** |
| **TG at baseline** | **-0.0028** | **-0.0055, -0.0001** | **0.026** | **24.95** | **59.11** | **0.04** |
| **TG at follow-up** | **-0.0037** | **-0.0081, 0.0007** | **0.030** | **13.96** | **63.14** | **0.10** |
| **TC at baseline** | **-0.0009** | **-0.0046, 0.0028** | **0.034** | **-0.63** | **66.77** | **0.62** |
| **TC at follow-up** | **-0.0031** | **-0.0071, 0.0010** | **0.030** | **9.84** | **61.10** | **0.13** |
| **Type pf drugs** | **0.0158** | **-0.0178, 0.0495** | **0.031** | **4.30** | **62.26** | **0.35** |
| **Dosage of drugs** | **0.0910** | **0.0046, 0.1774** | **0.027** | **19.52** | **53.84** | **0.04** |
| **Smoking** | **-0.0000** | **-0.0071, 0.0071** | **0.035** | **-3.17** | **70.73** | **0.99** |
| **Diabetes** | **-0.0007** | **-0.0084, 0.0071** | **0.031** | **-3.31** | **66.16** | **0.87** |
| **Hypertension** | **0.0010** | **-0.0040, 0.0059** | **0.033** | **-1.51** | **68.52** | **0.69** |

**PAV, percent atheroma volume; LDL, low-density lipoproteins; HDL, high-density lipoproteins; TG, triglycerides; TC, total cholesterol.**

**Table S4. Regression Analysis for TAV**

| **Variable** | **Coefficient** | **95% CI** | **tau^2^** | **Adj R-squared (%)** | **I-squared res (%)** | **P** |
| --- | --- | --- | --- | --- | --- | --- |
| **Age** | **-0.0093** | **-0.0276, 0.0089** | **0.021** | **-4.25** | **47.80** | **0.31** |
| **Gender** | **0.0086** | **0.0006, 0.0164** | **0.020** | **1.43** | **46.56** | **0.03** |
| **Region** | **0.0167** | **-0.0567, 0.0901** | **0.022** | **-3.51** | **46.15** | **0.65** |
| **LDL at baseline** | **-0.0018** | **-0.0057, 0.0022** | **0.019** | **5.61** | **41.33** | **0.38** |
| **LDL at follow-up** | **-0.0045** | **-0.0080, -0.0011** | **0.011** | **43.98** | **30.49** | **0.01** |
| **HDL at baseline** | **-0.0087** | **-0.0254, 0.0080** | **0.021** | **-7.02** | **48.01** | **0.30** |
| **HDL at follow-up** | **-0.0028** | **-0.0165, 0.0109** | **0.021** | **-6.82** | **47.31** | **0.69** |
| **TG at baseline** | **-0.0015** | **-0.0036, 0.0007** | **0.013** | **16.99** | **31.84** | **0.18** |
| **TG at follow-up** | **-0.0032** | **-0.0067, 0.0003** | **0.012** | **24.19** | **28.89** | **0.07** |
| **TC at baseline** | **-0.0012** | **-0.0039, 0.0014** | **0.014** | **6.54** | **35.15** | **0.35** |
| **TC at follow-up** | **-0.0022** | **-0.0051, 0.0006** | **0.012** | **21.70** | **27.85** | **0.13** |
| **Type pf drugs** | **0.0123** | **-0.0149, 0.0395** | **0.019** | **7.96** | **41.66** | **0.37** |
| **Dosage of drugs** | **0.0450** | **-0.0268, 0.1168** | **0.018** | **12.60** | **39.62** | **0.21** |
| **Smoking** | **0.0035** | **-0.0008, 0.0078** | **0.021** | **0.13** | **48.17** | **0.11** |
| **Diabetes** | **-0.0004** | **-0.0066, 0.0058** | **0.015** | **-3.81** | **34.09** | **0.90** |
| **Hypertension** | **0.0019** | **-0.0021, 0.0059** | **0.013** | **5.76** | **31.62** | **0.35** |

**TAV, total atheroma volume; LDL, low-density lipoproteins; HDL, high-density lipoproteins; TG, triglycerides; TC, total cholesterol.**

**eFigure 1.** PRISMA Flow Diagram

**eFigure 2.** Funnel Plots for included studies: (A) funnel Plots for TAV; (B) funnel Plots for PAV.

**eFigure 3.** Sensitivity analysis for included studies: (A) sensitivity analysis for TAV; (B) sensitivity analysis for PAV.

**eFigure 4.** Subgroup analysis for SMD in plaque volume between patients at baseline and follow-up: (A) subgroup analysis about TAV by the different drug administration; (B) subgroup analyses about PAV by the different drug administration.
