# Supplementary figures and images for: The effects of lipid-lowering therapy on coronary plaque regression: a systematic review and meta-analysis
Source: Sci Rep. 2021 Apr 12;11:7999. doi: 10.1038/s41598-021-87528-w (PMC8042107; doi:10.1038/s41598-021-87528-w)

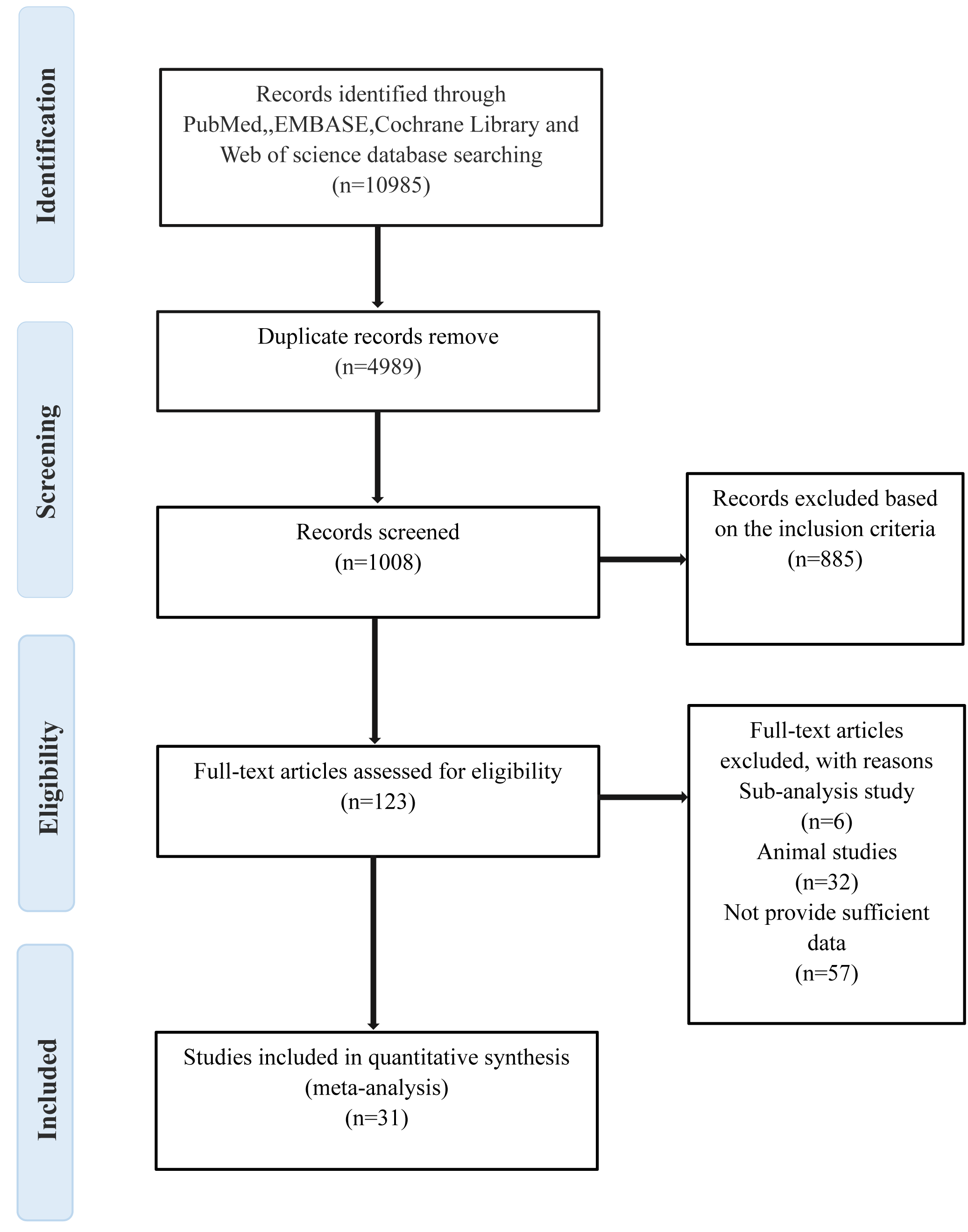

Supplement: Supplementary file 2 — Supplementary Figure S1. [file 41598_2021_87528_MOESM2_ESM.tif]

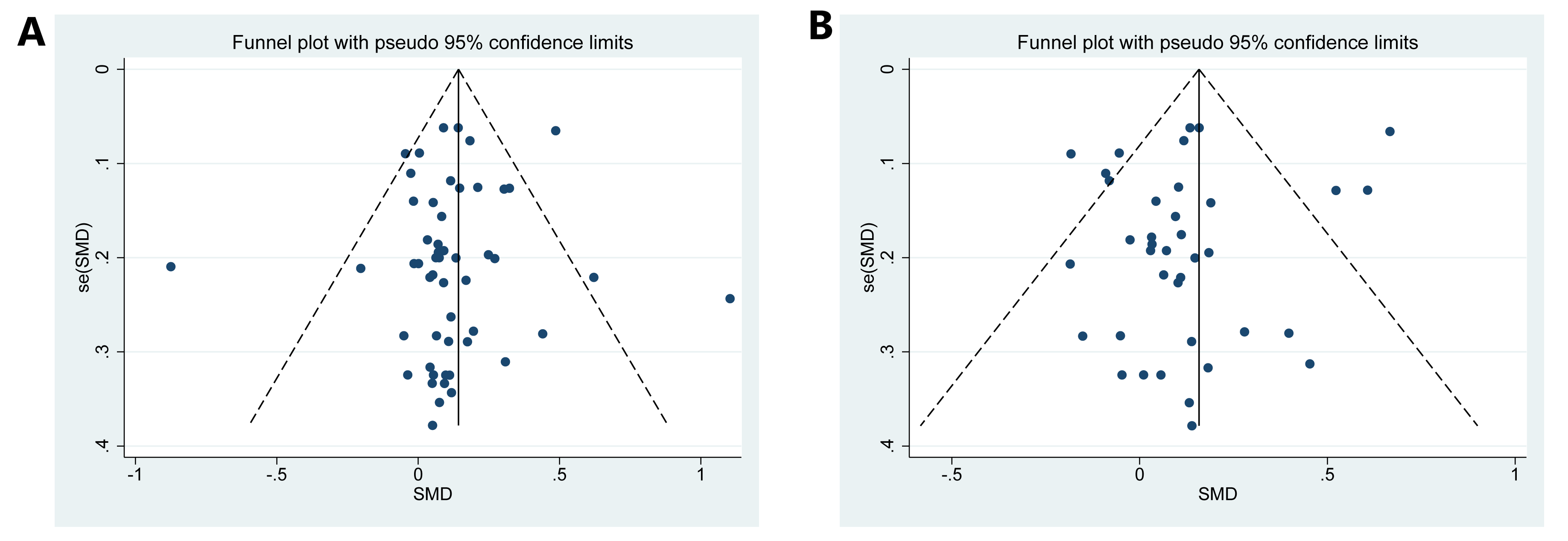

Supplement: Supplementary file 3 — Supplementary Figure S2. [file 41598_2021_87528_MOESM3_ESM.tif]

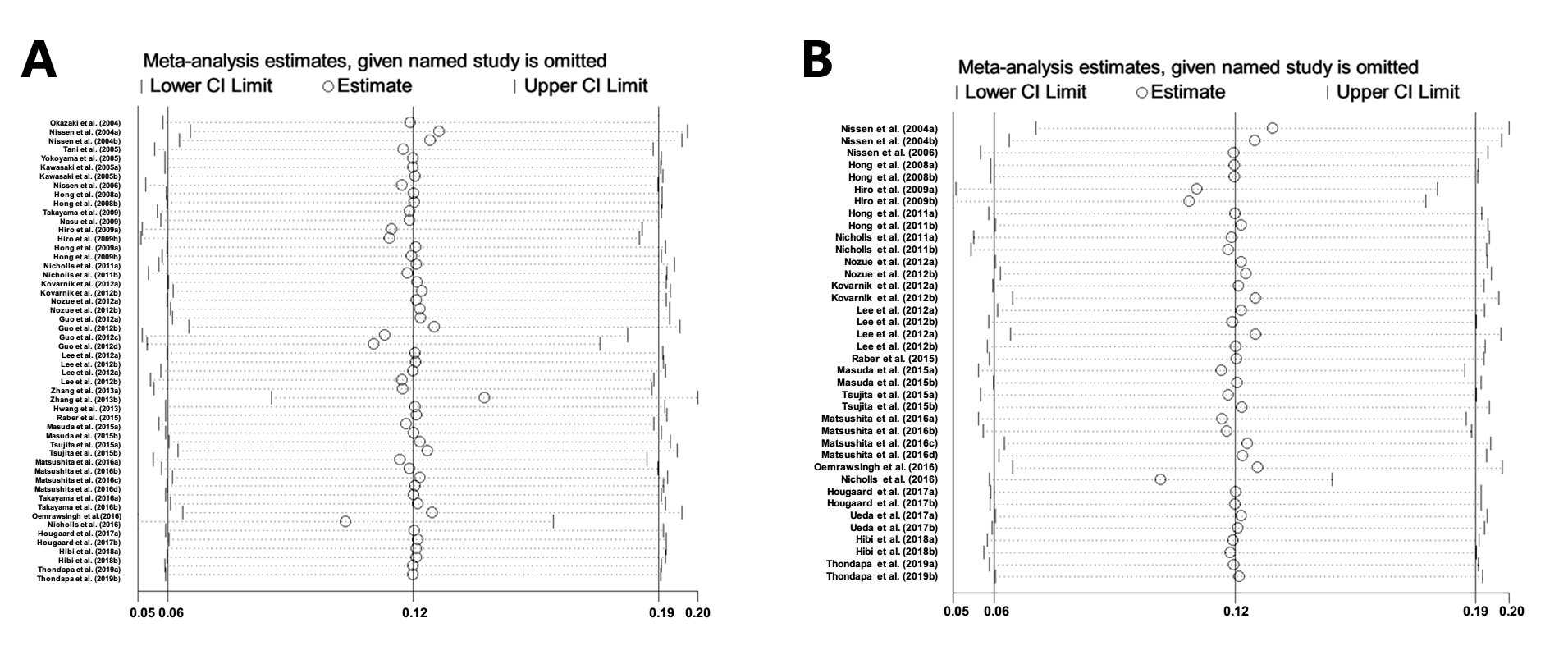

Supplement: Supplementary file 4 — Supplementary Figure S3. [file 41598_2021_87528_MOESM4_ESM.tiff]

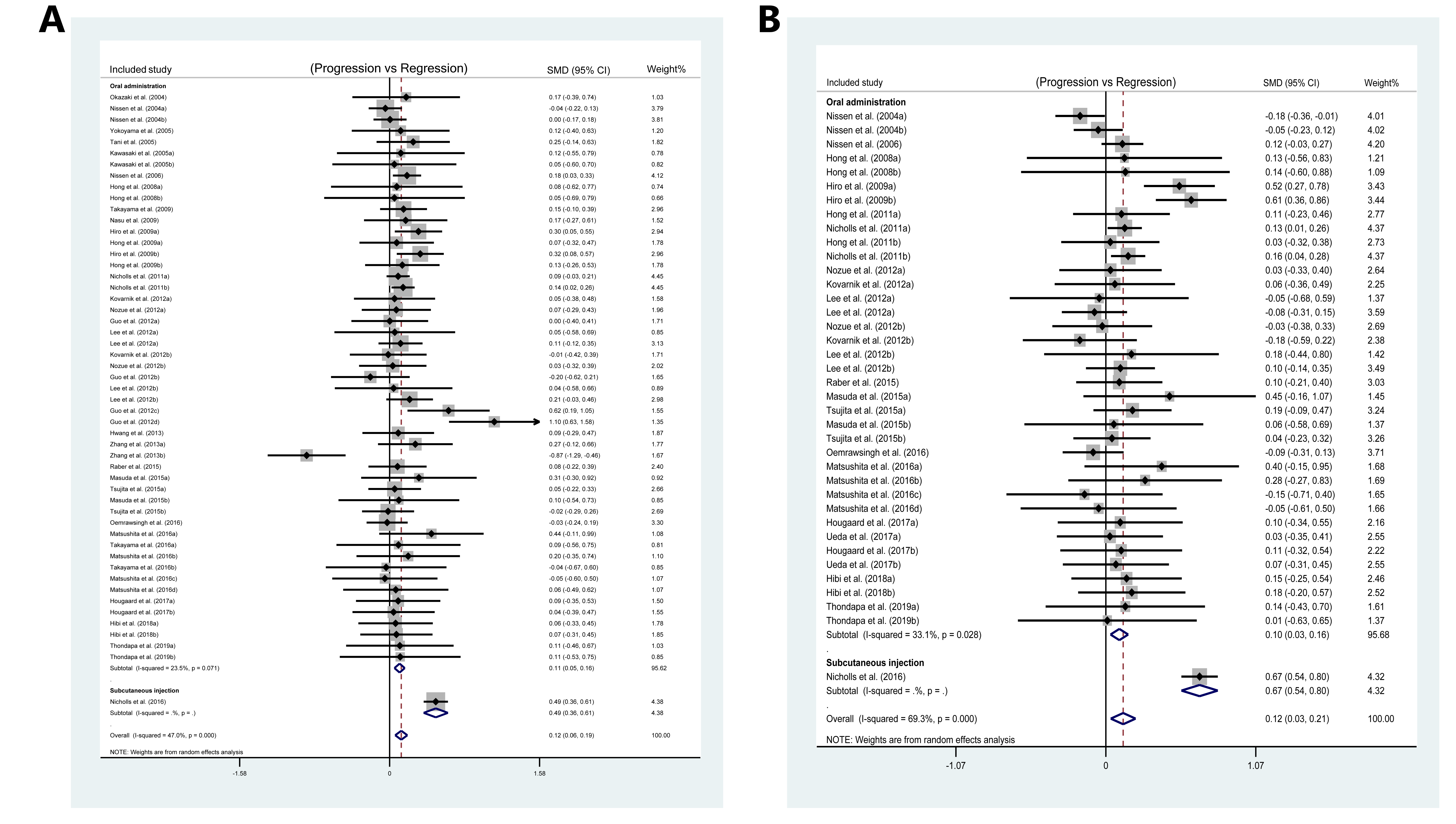

Supplement: Supplementary file 5 — Supplementary Figure S4. [file 41598_2021_87528_MOESM5_ESM.tif]
